# Somatic genome‐doubling is the most parsimonious route to allopolyploidy

**DOI:** 10.1111/nph.71179

**Published:** 2026-04-12

**Authors:** Robin Burns, Alison Dawn Scott, Polina Yu Novikova

**Affiliations:** ^1^ Department of Plant Sciences University of Cambridge Cambridge CB23EA UK; ^2^ Department of Chromosome Biology Max Planck Institute for Plant Breeding Research Cologne D‐50829 Germany; ^3^ Department of Plant Biotechnology and Bioinformatics Ghent University Technologiepark 71 Ghent 9052 Belgium; ^4^ VIB Center for Plant Systems Biology Technologiepark 71 Ghent 9052 Belgium

**Keywords:** genome evolution, hybridization, meiosis, polyploidy, somatic mutation

## Abstract

The origin of a polyploid can hinge on a single errant cell division, a mistake in the cell cycle that leads to genome‐doubling and re‐writes the rules of chromosome pairing and segregation. In plants, the evolutionary significance of these errors is magnified by lack of an early sequestered germline, meaning somatic mutations can be heritable. For sterile hybrids, somatic genome‐doubling can be an immediate beneficial mutation, providing each chromosome with a homolog for pairing during meiosis, partially restoring fertility. This review examines evidence for the origin of polyploid hybrids, known as allopolyploids. While the involvement of unreduced gametes or triploid bridges remains plausible, both natural and synthetic allopolyploids indicate that somatic genome‐doubling following hybridization is the most parsimonious route.

**Table:** 

	Contents	
	Summary	2845
I.	Introduction	2845
II.	Polyploidization‐first route	2846
III.	Hybridization‐first route	2847
IV.	On the importance of triploid bridges	2847
V.	Evolutionary reconstructions of allopolyploid origins	2847
VI.	The propensity for allopolyploidization	2848
VII.	Conclusion	2848
	Acknowledgements	2848
	References	2849

## I. Introduction

Polyploidization (whole‐genome duplication, WGD) has long been recognized as a major driver of plant genome evolution and has even been proposed as a solution to Darwin's famous ‘abominable mystery’ surrounding the rapid radiation of angiosperms (Darwin, 1903; MacKintosh & Ferrier, 2017). Hybrid polyploids, or allopolyploids, are especially interesting, as they provide a natural experiment in which two divergent genomes, with their complete chromosome set, are suddenly forced to co‐exist in a single nucleus. Despite decades of study and agricultural interest in allopolyploids (Mason & Batley, 2015; Akagi *et al*., 2022; Li *et al*., 2024), the precise routes by which new allopolyploids originate remain to be fully resolved.

**Fig. 1:**
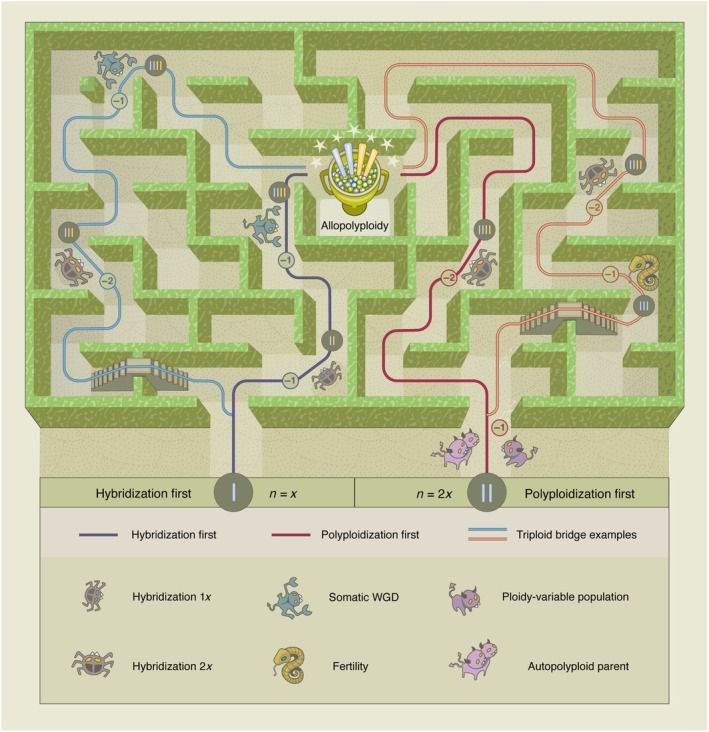
Alternative routes to allopolyploid origin. The origin of an allopolyploid can be conceptualized as alternative routes through a maze, each comprising a sequence of rare or unfavorable events. The hybridization‐first route (blue) begins with homoploid hybridization (*n* = *x*) followed by somatic whole‐genome duplication (WGD), whereas the polyploidization‐first route (red) begins with genome duplication (*n* = 2*x*) before interspecific hybridization and typically involves autopolyploid parents, which are natural sources of 2*x* gametes. Detours representing triploid bridge scenarios branch from both main routes (light blue and light red). Unfavorable steps are depicted as monsters, symbolizing selective, mutational, or demographic barriers that reduce the probability of successful progression through the maze. The most parsimonious route, encountering the fewest monsters, is the hybridization‐first pathway involving hybridization followed by somatic WGD. By contrast, the polyploidization‐first route incurs an initial barrier because it requires a nonstandard (2*x*) gamete, produced either as an unreduced gamete or by an autotetraploid parent. Gametes from autotetraploids face additional barriers owing to altered crossover landscapes and increased mutation loads. Triploid bridges represent longer and less likely detours. In the hybridization‐first route, a triploid bridge (light blue) can arise through hybridization involving a 2*x* gamete, producing a potentially fertile allotriploid. Clonal propagation followed by somatic WGD represents one possible route to a fertile allotetraploid without gamete involvement, thereby avoiding a fertility monster. In the polyploidization‐first route, a triploid bridge (light red) can arise from autotetraploid *x* diploid crosses in mixed‐ploidy populations, producing an autotriploid. Formation of an allotetraploid then requires autotriploid fertility and hybridization with non‐standard gametes from another species, further increasing the cumulative number of monsters encountered. Triploid bridges shown are simplified examples, and only allotetraploid outcomes are illustrated, higher order allopolyploids (e.g. allohexaploids) are also possible.

The origin of new allopolyploids can be viewed as a two‐step process: the merging of divergent genomes (hybridization) and the doubling of the chromosome set (polyploidization). Either step can occur first, producing two alternative routes (Fig. 1). In the polyploidization‐first route, genome doubling in the parental germline produces unreduced (2*n*) gametes that later fuse. In the hybridization‐first route, a typically sterile hybrid regains fertility when somatic genome doubling restores homologous pairing. Although both routes lead to a fertile allopolyploid, they differ in their genetic mechanisms and prerequisites.

## II. Polyploidization‐first route

The hallmark of the polyploidization‐first route is that WGD occurs before hybridization (Fig. 1). Although 2*n* gametes occur across species (Bretagnolle & Thompson, 1995; Ramsey, 2007; Kreiner *et al*., 2017), their simultaneous production and hybridization are unlikely. The route is more plausible when one or both progenitors are autotetraploid, as their reduced gametes are compatible with diploid 2*n* gametes. Synthetic allopolyploids derived from crosses of autopolyploids are possible (Chen *et al*., 1998; Burns *et al*., 2021; Chéron *et al*., 2023), though aneuploidy is frequent (Burns *et al*., 2021).

Sugarcane (*Saccharum* spp.) is an example of this route. Early 1900s crosses between the autopolyploids *S. officinarum* and *S. spontaneum* produced hybrids in which *S. officinarum* contributed an unreduced female gamete (2*n*), while *S. spontaneum* contributed a normal male gamete (n), producing 2*n* + *n* offspring. Repeated backcrossing to *S. officinarum* restored n + n inheritance and progressively diluted the *S. spontaneum* genome (Vieira *et al*., 2018). Modern cultivars carry ~75–85% *S. officinarum* chromosomes and 15–25% *S. spontaneum* (Healey *et al*., 2024; Zhang *et al*., 2025). Despite autopolyploid parentage, cultivars show unstable karyotypes (Vieira *et al*., 2018).

The involvement of autopolyploid parents in the polyploidization‐first route may lead to genome instability in neoallopolyploids for several reasons. Neoautopolyploids have meiotic irregularities and low fertility (Yao *et al*., 2011; Westermann *et al*., 2024), and must adapt to suppress multivalents, involving synaptonemal complex genes (Hollister *et al*., 2012; Yant *et al*., 2013; Bohutínská *et al*., 2021) in some cases or the kinetochore (Bray *et al*., 2024) in others. By contrast, neoallopolyploids are typically disomic from the outset (Pecinka *et al*., 2011; Roux & Pannell, 2015; Li *et al*., 2021) but must suppress homeologous recombination (Henry *et al*., 2014; Higgins *et al*., 2021; Serra *et al*., 2021). Established autopolyploids (e.g. *Arabidopsis arenosa*, *Arabidopsis lyrata*, *Cochlearia*) have reduced crossovers and altered interference (Comai *et al*., 2003; Yant *et al*., 2013; Morgan *et al*., 2021), while allopolyploids (e.g. wheat, *Brassica napus*, and *Arabidopsis suecica*) adapt DNA‐repair pathways to prevent homeologous pairing (Rey *et al*., 2017; Higgins *et al*., 2021; Serra *et al*., 2021; Burns *et al*., 2026). The contrasting meiotic adaptations between the ploidy types likely hinders stabilization (Vieira *et al*., 2018; Burns *et al*., 2021; Chéron *et al*., 2023). Additionally, deleterious mutation load in autopolyploids (Baduel *et al*., 2019; Vlček *et al*., 2025) can be exposed in self‐compatible neoallopolyploids, increasing extinction risk despite homeolog buffering (Burns *et al*., 2026).

## III. Hybridization‐first route

The hybridization‐first route begins with fusion of normal gametes from diploid progenitors (Fig. 1). Since diploids are more frequent than polyploids (Rice *et al*., 2019), hybridization between diploids is also likely more frequent. Importantly, divergence in genome sequence and karyotype can result in hybrid sterility (Stathos & Fishman, 2014; Marta *et al*., 2023). In plants, where hybridization is common (Mallet, 2005), and the germline is not sequestered early, somatic genome doubling can be a heritable mutation. By providing homologs for meiotic pairing, somatic WGD offers an immediate solution to sterility.

Several natural cases illustrate this route. *Sporobolus anglicus* (2*n* = 4*x* = 120–124) originated in the nineteenth century (Lambert, 1964; Marchant, 1968; Salmon *et al*., 2025) and is likely a doubled derivative of the clonal, sterile diploid hybrid *S*. ✕ *townsendii* (Marchant, 1963; Raybould, 1998). In monkeyflowers, the fertile allohexaploid *Mimulus peregrinus* (2*n* = 6*x* = 92) (Vallejo‐Marin, 2012; Vallejo‐Marín *et al*., 2015) similarly appears to have originated via somatic doubling of the clonal sterile triploid hybrid *M*. ✕ *robertsii* (Vallejo‐Marin & Lye, 2012). The allotetraploid fern *Asplenium tutwilerae* (2*n* = 4*x* = 144) is likely derived from genome‐doubling of the clonal, sterile diploid hybrid *A*. × *ebenoides* (Wagner, 1957). While not observed directly, somatic WGD is the most plausible event that restored fertility in these clonally persistent, sterile hybrids.

A classic example of fertility restored by somatic WGD is *Primula kewensis*, derived from a sterile diploid hybrid of *P. floribunda* and *P. verticillata* (both 2*n* = 2*x* = 18). Rare fertile inflorescences produced seeds yielding allotetraploids (2*n* = 4*x* = 36), confirmed cytologically. Selfed progeny show variable karyotypes (2*n* = 34–37) (Newton & Pellew, 1929), highlighting that bivalent formation in neoallopolyploids does not guarantee stable meiosis.

Laboratory crosses in *Arabidopsis* provide another real‐time example. Crosses between *A. thaliana* (2*n* = 2*x* = 10) and *A. lyrata* (2*n* = 2*x* = 16) produce sterile F_1_ hybrids unable to complete meiosis. In one of five hybrids, self‐fertile inflorescences appeared and produced fertile allopolyploids. Cytology showed these plants underwent somatic WGD, restoring meiosis (Nasrallah *et al*., 2000).

## IV. On the importance of triploid bridges

When the formation of triploids is not hindered by the triploid block, a postzygotic barrier due to parental genome imbalance (Köhler *et al*., 2021) triploid intermediates can provide an alternative route, or bridge, to allopolyploidy (Köhler *et al*., 2010) (Fig. 1). Interploidy gene flow is documented in several plant species (Bartolić *et al*., 2024; Brown *et al*., 2024). Autotriploids can, if fertile, produce 2*n* gametes, but evidence for their stability is mixed. In *A. arenosa*, autotriploids are virtually absent from contact zones (Morgan *et al*., 2020). In *Chamerion angustifolium* and *Cardamine amara*, rare triploids occur but have low fertility (Husband, 2004; Bartolić *et al*., 2025). These observations indicate that while autotriploids can occasionally mediate gene flow, they are an unreliable route to allopolyploidy.

Allotriploids are typically sterile, requiring WGD to restore fertility, for example in the allohexaploid *M. peregrinus*. In principle, an allotetraploid could arise from an allotriploid via hybridization involving 2*n* gametes or via somatic WGD, but both scenarios require additional steps and partial genome loss, much less parsimonious than simple doubling to an allohexaploid. In *B. napus*, synthetic allotriploids have yielded fertile allotetraploids (Cao *et al*., 2023), possibly from unreduced gametes though somatic WGD could also contribute. Trisomic maize lines show reduced vigor (Yang *et al*., 2025). In *Populus*, natural allotriploids show strong meiotic instability and low pollen viability (Wang *et al*., 2017). Many natural stable allotriploids persist asexually (Mock *et al*., 2012; Kolář *et al*., 2017; Brukhin *et al*., 2019; Zozomová‐Lihová *et al*., 2025). Overall, the available evidence provides little support for a reliable sexual triploid bridge.

## V. Evolutionary reconstructions of allopolyploid origins

While recent case studies have documented the leap from sterile hybrids to fertile allopolyploids through direct observations, evolutionary older events can be reconstructed from population genomic data. Population genomic comparisons frequently identify extant diploid populations, rather than autopolyploids, as the genetically closest relatives of allopolyploids, supporting somatic genome‐doubling as the mechanism of allopolyploid origin. Population‐level sampling of the polyploid and its progenitors is essential for successful origin reconstruction. Examples in *Brassicaceae* also reveal recurrent shifts to self‐compatibility. Self‐compatibility provides reproductive assurance, and this greatly facilitates species establishment. Together, the evidence shows that the hybridization‐first route is frequent in allopolyploids.

In *Arabidopsis*, two natural allopolyploid species are known, *A. suecica* and *A. kamchatica*. The origin of both allopolyploid species has recently been fully resolved through population‐level sequencing of their progenitors. *Arabidopsis suecica* (2*n* = 4*x* = 26) is a post‐glacial polyploid hybrid derived from multiple crosses between *A. thaliana* (maternal) and *A. arenosa* (paternal) and is endemic to Fennoscandia (Novikova *et al*., 2017; Burns *et al*., 2021; Chéron *et al*., 2023). The closest *A. thaliana* accessions to *A*. *suecica* lie in Central Eurasia (Novikova *et al*., 2017; Burns *et al*., 2026), but until recently, the cytotype of the *A. arenosa* parent was unknown. Autotetraploid *A. arenosa* populations are widespread in Europe (Monnahan *et al*., 2019), raising the possibility of a polyploidization‐first route involving the fusion of an unreduced *A. thaliana* gamete with a normal gamete from autotetraploid *A. arenosa* (Jakobsson *et al*., 2006). However, genomic data ruled this hypothesis out, identifying diploid *A. arenosa* as the parent (Burns *et al*., 2024), supporting a hybridization‐first route. Selection scans also showed *de novo* adaptation in meiotic genes, consistent with additional adaptations (Burns *et al*., 2024) being required to fully restore fertility (Chéron *et al*., 2023). *Arabidopsis* *suecica* was likely self‐compatible since origin as the *A. thaliana* subgenome carries a dominant nonfunctional *S*‐allele capable of silencing the functional *S*‐alleles inherited from *A*. *arenosa* (Novikova *et al*., 2017, 2023).

*Arabidopsis kamchatica* (2*n* = 4*x* = 32) originated from multiple hybridization events between *A. halleri* (maternal) and *A. lyrata* (paternal) around the Japanese archipelago and the Kamchatka Peninsula, later expanding into northeast Russia and northwestern North America (Shimizu‐Inatsugi *et al*., 2009; Kolesnikova *et al*., 2023). Population genomic analyses have refined the origin, showing that the closest relatives of the *A. lyrata* subgenome of *A. kamchatica* are descended from a diploid self‐compatible Siberian lineage (Kolesnikova *et al*., 2023). This finding in *A. kamchatica*, like in *A. suecica*, rules out a polyploidization‐first route, favoring hybridization‐first; and further indicates that the neoallotetraploid was self‐compatible from the outset, inherited from the *A. lyrata* progenitor.

The origin of *Capsella bursa‐pastoris* is also well‐resolved. Since no autotetraploids exist in the genus, a polyploidization‐first route is unlikely. Genomic evidence shows *C. bursa‐pastoris* originated via hybridization between *C. orientalis* (maternal) and an outcrossing *C. grandiflora/C. Rubella* lineage (Douglas *et al*., 2015; Bachmann *et al*., 2021). Experimental crosses yield sterile diploid hybrids that can spontaneously double, restoring fertility and are self‐compatible (Bachmann *et al*., 2021). Comparisons of the two routes indicate higher fertility in hybridization‐first individuals that is likely due to deleterious mutation load in the autopolyploid progenitors (Duan *et al*., 2023, 2024).

Outside of Brassicaceae, the cultivated peanut *Arachis hypogaea* illustrates the hybridization‐first route. *Arachis hypogaea* diploid progenitors – *A. duranensis* and *A. ipaensis* – hybridized roughly 10 000 yr ago (Bertioli *et al*., 2020), likely producing a sterile hybrid because experimental crosses are sterile (Leal‐Bertioli *et al*., 2021). Genomic evidence points to a narrow, likely single origin (Bertioli *et al*., 2019), supporting the idea that somatic genome‐doubling restored fertility in a sterile hybrid (Leal‐Bertioli *et al*., 2021). Like *Arabidopsis* and *Capsella*, peanut is self‐compatible, which likely facilitated its establishment.

These reconstructions support the hybridization‐first route; however, they are concentrated in *Brassicaceae*, and more studies will be needed to resolve how broadly the route applies.

## VI. The propensity for allopolyploidization

A central question is whether sterile hybrids have a propensity for somatic genome doubling, either from inherited alleles or from cell‐division errors due to genome divergence, or both? Since sterile hybrids likely are short‐lived, we mainly observe those rescued by genome doubling. Direct measurements of mitotic error rates in hybrids vs their progenitors are scarce, but crucial for quantifying predisposition to WGD. Somatic mutations in *Arabidopsis* are 2‐7*x* that of germline (Meyer *et al*., 2025). In yeast, seven out of 600 sterile hybrids regained fertility through spontaneous WGD within *c*. 400 mitotic divisions, demonstrating that fertility can be restored before sexual reproduction (Charron *et al*., 2019).

Direct evidence for alleles that drive heritable somatic genome‐doubling in mitotic cells is lacking. However, endoreduplication studies identify genes that modulate somatic ploidy in *A. thaliana*: CCS52 promotes mitotic exit and repeated endocycles (Cebolla *et al*., 1999; Vanstraelen *et al*., 2009). SIAMESE triggers endoreduplication and suppresses mitosis (Churchman *et al*., 2006), and DP‐E2F‐like represses the endocycle, with mutants showing elevated ploidy (Vlieghe *et al*., 2005). These findings suggest that natural alleles affecting somatic WGD likely exist; the challenge is to determine how they act in mitotically active meristems.

## VII. Conclusion

The evidence across natural, synthetic, and agricultural allopolyploids points to a common pattern in their origin: hybridization first, somatic genome‐doubling second, and self‐compatibility often as the final piece that facilitates species establishment.

Why is allopolyploidy ubiquitous in plants? Part of the answer may be explained by the fact that plant speciation is not a simple bifurcation (Novikova *et al*., 2016; Filiault *et al*., 2018) and is better described as a reticulate network (Mallet *et al*., 2016), in which hybridization between species is recurrent. Alleles promoting somatic genome doubling can drift in diploids yet become strongly favored in sterile hybrids because they partially restore fertility. Plants do not just tolerate polyploidy; somatic genome doubling is a repeated solution to overcome hybrid sterility.

## Competing interests

None declared.

## Disclaimer

The New Phytologist Foundation remains neutral with regard to jurisdictional claims in maps and in any institutional affiliations.
